# Determinants of interactions of a novel next-generation gabapentinoid NVA1309 and mirogabalin with the Cavα2δ-1 subunit

**DOI:** 10.1186/s13041-024-01129-y

**Published:** 2024-08-07

**Authors:** Ivana A. Souza, Maria A. Gandini, Md Yousof Ali, Franz Kricek, George Skouteris, Gerald W. Zamponi

**Affiliations:** 1 Department of Clinical Neurosciences, Cumming School of Medicine, Hotchkiss Brain Institute, Alberta Children’s Hospital Research Institute, University of Calgary, Calgary, AB Canada; 2 Department of Experimental Neurosciences, Novassay SA, Biopôle, 1066 Epalinges, Switzerland; 3 NBS-C BioScience GmbH, 1230 Vienna, Austria; 4 3A Laboratories, Stevenage Bioscience Catalyst (SBC), Stevenage, SG1 2FX UK

**Keywords:** Mirogabalin, Gabapentinoids, Calcium channel, Neuropathic pain, Cavα2δ

## Abstract

NVA1309 is a non-brain penetrant next-generation gabapentinoid shown to bind Cavα2δ at R243 within a triple Arginine motif forming the binding site for gabapentin and pregabalin. In this study we have compared the effects of NVA1309 with Mirogabalin, a gabapentinoid drug with higher affinity for the voltage-gated calcium channel subunit Cavα2δ-1 than pregabalin which is approved for post-herpetic neuralgia in Japan, Korea and Taiwan. Both NVA1309 and mirogabalin inhibit Cav2.2 currents in vitro and decrease Cav2.2 plasma membrane expression with higher efficacy than pregabalin. Mutagenesis of the classical binding residue arginine R243 and the newly identified binding residue lysine K615 reverse the effect of mirogabalin on Cav2.2 current, but not that of NVA1309.

**Supplementary Information:**

The online version contains supplementary material available at 10.1186/s13041-024-01129-y.

Mirogabalin is a novel gabapentinoid drug developed for the treatment of neuropathic pain. Like gabapentin (GBP) and pregabalin (PGB), it selectively binds to the Cavα2δ-1 and Cavα2δ-2 subunits of voltage-gated calcium channels (VGCCs) (1). It is believed that gabapentinoid binding to Cavα2δ disrupts VGCC trafficking to the plasma membrane, leading to decreased transmission of nociceptive information (2). Mutagenesis of the Cavα2δ structure has revealed an arginine at position R243 (R241 in the human protein, see Supplemental Methods) that is crucial for GBP and PGB binding (3, 4). This arginine, however, must be embedded within the structurally intact region of Cavα2δ for proper binding of these drugs. Recently, it was shown that mirogabalin also binds to the same region within the extracellular dCache_1 domain. Comprehensive Ala-scanning mutagenesis analysis identified 12 important residues: Y236, R241, W243, Y450, D452, T461, D491, W205, V207, Y217, W223, and L454 (in human sequence) (5). Kitano et al. (6) previously showed that 50 µM mirogabalin decreases N-type channel currents in cultured rat dorsal root ganglion neurons.

We recently reported functional effects of a novel, non-brain penetrant gabapentinoid, NVA1309, on Cav2.2 N-type calcium channel trafficking and function (7). Target binding experiments using Surface Plasmon Resonance (SPR) showed that, in contrast to GBP, NVA1309 is still able to bind to R243 in truncated Cavα2δ constructs, revealing a unique interaction mechanism. We also identified a second amino acid sequence (IKAKLEETITQA) containing lysine residue K615 as a critical binding hot spot within the carboxy-terminal part of the VGCC-α2 domain, but K615 did not appear to be involved in the functional effects of NVA1309 on Cav2.2 (7). Here, we expand on our results and compare the effects of mirogabalin to prior findings with NVA1309 (7) on Cav2.2 trafficking and function and use molecular docking to gain insights into their interactions with Cavα2δ.

To analyse the effect of 100 µM of mirogabalin on heterologously expressed Cav2.2 channels, tsA201 cells were transfected with Cav2.2, Cavβ1 and wild-type or mutated Cavα2δ-1 (R243A, K615A or double mutant R243A + K615A). Cells were treated for 48 h with either DMSO or 100 µM mirogabalin before recording whole cell barium currents as described in (7). Figure 1a reveals a statistically significant mirogabalin-mediated reduction in current density compared to DMSO in cells expressing wild-type Cavα2δ-1. The effect of mirogabalin on Cav2.2 currents was lost in channels expressed with Cavα2δ-1 R243A, K615A and R243A + K615A, respectively (Figs. 1b–d**)**, suggesting that, in addition to R243, K615 is also important for mirogabalin interactions with Cavα2δ-1. This differs from our prior results with NVA1309 where mutation of residue K615 had no effect (7).

**Fig. 1:**
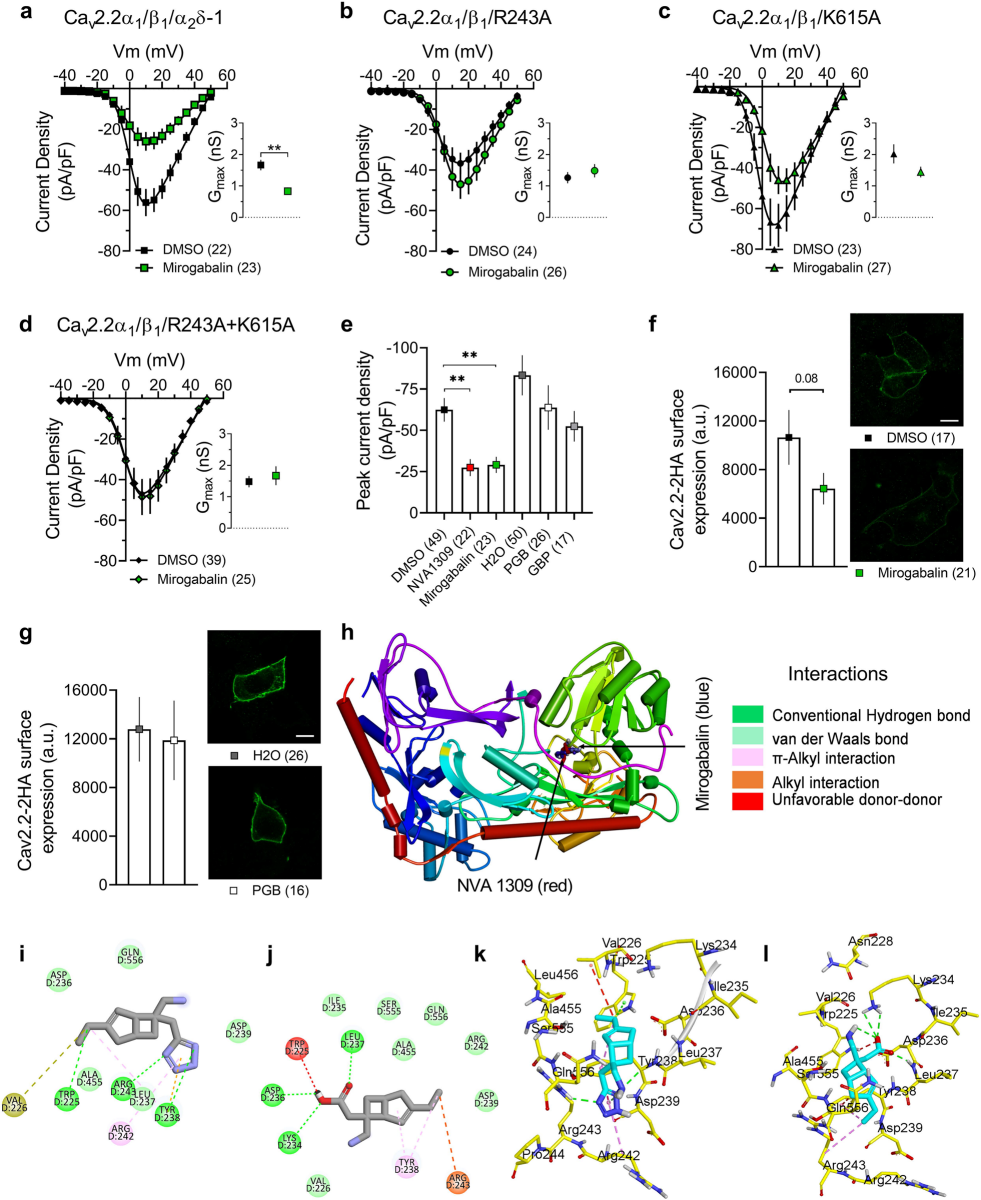
Effect of mirogabalin on Cav2.2 function and trafficking. **a** Current density–voltage (I/V) relationships of cells co-expressing Cav2.2 with wild-type Cavα2δ-1 and treated with 100 μM NVA1307 for 48 h. The inset shows maximum conductance (**p = 0.0035, Mann–Whitney test). **b** Current density–voltage (I/V) relationships of cells expressing Cav2.2 with R243A Cavα2δ-1 and treated with 100 μM mirogabalin for 48 h. The inset shows maximum conductance. **c** Current density–voltage (I/V) relationships of cells co-expressing Cav2.2 with K615A Cavα2δ-1 and treated with 100 μM mirogabalin for 48 h. The inset shows maximum conductance. **d** Current density–voltage (I/V) relationships of cells expressing Cav2.2 with R243A + K615A Cavα2δ-1 and treated with 100 μM mirogabalin for 48 h. The inset shows maximum conductance. **e** Average peak current density from cells treated with 100 µM of NVA1309 (data from Ref. (7)), mirogabalin, PGB or GPB for 48 h. (**p = 0.0028 for NVA1309 and **p = 0.0098 for mirogabalin compared to DMSO, Kruskal–Wallis test followed by Dunn’s multiple comparisons test). **f** Effect of mirogabalin on membrane expression of Cav2.2HA after 48 h. **g** Effect of PGB on membrane expression of Cav2.2HA after 48 h. Fluorescence intensity was quantified using Image J software. Scale bar: 10 µm. **h** Overlapping of the docked NVA1309 (red) and mirogabalin (blue) within the full-length structure of Cavα2δ-1. **i **Predicted 2D NVA1309 interactions in the Cavα2δ-1 binding pocket. **j** Predicted 2D mirogabalin interactions in the Cavα2δ-1 binding pocket. **k** Predicted 3D NVA1309 interactions with Cavα2δ-1. **l** Predicted 3D mirogabalin interactions with Cavα2δ-1. Interactions are represented by green (conventional hydrogen bonding), light green (van der Waals bond), pink (π-alkyl interactions), orange (alkyl interactions), and red (unfavorable donor-donor)

By using SPR we compared kinetic constants and binding affinities of mirogabalin and NVA1309, applied as analytes for binding to a chemically synthesized peptide surrounding the K615 locus (IKAKLEETITQA; peptide P3 in (7)), immobilized on a Biacore optical sensor chip. Sensorgrams from serial dilutions were analysed by fitting with a Langmuir 1:1 interaction algorithm (BiaEvaluation 4.1 software). Kinetic binding constants were as follows: mirogabalin: k_a_ [1/Ms] = 14.2 ± 7.3; k_d_ [1/s] = 1.9 ± 0.6 E^−3^; K_D_ = 161 ± 60 µM; NVA1309: k_a_ [1/Ms] = 8.7 ± 2.77; k_d_ [1/s] = 2.1 ± 0.2 E^−3^; K_D_ = 273 ± 101 µM. The two-fold difference in K_D_ of the two compounds was, however, not statistically significant. We then compared the electrophysiological effects of 100 µM of multiple gabapentinoids on Cav2.2 average peak current density after 48 h treatment (Fig. 1e). Mirogabalin and NVA1309 significantly decreased average peak current density when compared to DMSO (**p = 0.0098 and **p = 0.0028 respectively, Kruskal–Wallis’ test followed by Dunn’s multiple comparisons test). While pregabalin (PGB) and gabapentin (GBP) seemed to mediate a small reduction in peak current densities, they were not statistically different from control. These data indicate that mirogabalin and NVA1309 are more effective than PGB or GBP in disrupting Cav2.2 channel activity when applied at 100 µM concentrations.

To examine effects on Cav2.2 trafficking, we analysed plasma membrane expression of these channels by immunocytochemistry using a Cav2.2channel with an external double hemagglutinin (HA) tag in tsA201 cells treated with either vehicle, 100 µM mirogabalin or pregabalin (PGB). Consistent with the effect previously observed with NVA1309 (7), chronic treatment with mirogabalin appeared to decrease Cav2.2HA membrane expression, however this was not statistically significant (*p = 0.08, Mann Whitney test) (Fig. 1f), differing with previous findings testing NVA1309 which were statistically significant (7). 100 µM PGB had no effect (Fig. 1g), consistent with the electrophysiological analysis. For comparison, a previous study reported decreased Cavα2δ-1 plasma membrane expression in COS-7 cells expressing Cav2.2 and treated for 72 h with 20 and 200 µM PGB (8).

We next performed molecular docking simulations using AutoDockVina (Supplemental methods). NVA1309 (red) and mirogabalin (blue) docking is displayed in Fig. 1h. NVA1309 and mirogabalin had a predicted binding affinity of − 6.3 and − 6.3 kcal/mol, respectively, for Cavα2δ-1. The NVA1309 tetrazole ring was predicted to form two important hydrogen bond interactions with Arg243 and Tyr238 in Cavα2δ-1 and forms a π-alkyl interaction with Arg242 and alkyl interactions with Tyr238. The ethyl group of NVA1309 establishes a hydrogen bond with Trp225 **(**Fig. 1i**)** and a π-alkyl interaction with Tyr238 and alkali interactions with Val226 in Cavα2δ-1. Next, we docked mirogabalin to Cavα2δ-1 and found three key hydrogen bond interactions with Asp236, Leu237, and Lys234 in the Cavα2δ-1. Additionally, mirogabalin was predicted to form a π-alkyl interaction withTyr238, alkyl interactions with Arg243, and unfavorable donor-donor interaction with Trp225 (Fig. 1j) 3D interactions are also provided for better understanding of the pocket site residues of NVA1309 and mirogabalin in the Cavα2δ-1 (Fig. 1k, and l). Cyro-EM data revealed that GBP occupies a pocket formed by Trp225, Tyr238, Arg243, and Ala455 (9), indicating a similar binding pattern compared to mirogabalin and NVA1309. Docking analysis did not reveal physical interaction of either compound with K615, although both are capable of specifically binding to isolated peptide sequences flanking K615. Thus, modulation of mirogabalin effects on channel trafficking by K615 mutants by might occur via sterical rearrangement in full length Cavα2δ-1.

The poor efficacy of current neuropathic pain treatments highlights the need of novel therapies (10). Mirogabalin showed a better balance between efficacy and safety in clinical trials using a step-wise titration regimen compared to pregabalin in patients with peripheral chronic neuropathic pain. While well tolerated in patients with post-herpetic neuralgia and diabetic neuropathy pain, adverse effects such as somnolence, dizziness, weight gain and suicidal ideation were reported (11). Mirogabalin and NVA1309 appear to share similar binding properties and effects on Cav2.2 channels. However, since NVA1309 is peripherally restricted, this compound has the potential of having a better side effect profile compared to Mirogabalin, PGB and GBP. This will however need to be confirmed in clinical studies.

### Supplementary Information

Additional file 1.

## Data Availability

All data generated or analysed during this study are included in this published article.

## Abbreviations

VGGC: Voltage-gated calcium channels
PGB: Pregabalin
GBP: Gabapentin
SPR: Surface Plasmon Resonance
