# Supplementary material for: Determinants of interactions of a novel next-generation gabapentinoid NVA1309 and mirogabalin with the Cavα2δ-1 subunit
Source: Mol Brain. 2024 Aug 7;17:54. doi: 10.1186/s13041-024-01129-y (PMC11308618; doi:10.1186/s13041-024-01129-y)
Supplement: Supplementary file 1 — Additional file 1. [file 13041_2024_1129_MOESM1_ESM.pdf]

SUPPLEMENTARY MATERIALS AND METHODS

Expression constructs and Cavα2δ numbering

The numerical position of the arginine and lysine amino acids that are important for gabapentinoid binding depends on the species and splice isoform of Cavα2δ-1 being referred to. For clarity, we use R243 in the figure, which corresponds to the third arginine in the RRR motif in the Cavα2δ-1 sequence used for molecular docking. In our previous study, it was referred to as R241. Lysine 615 corresponds to the canonical rat sequence used in the electrophysiology experiments and is in the 634 position in human sequence, which was used for SPR experiments.

Molecular docking analysis

The cryo-EM structures of the human L-type voltage gated calcium channel Ca_V1.2- Cavα2δ-1 complex were obtained from the RCSB Protein Data Bank (PDB ID: 8EOG) [1]. The protein was prepared using Accelrys Discovery Studio 19.1 (Accelrys, San Diego, CA, USA) [2]. The reported binding area between co-ligand and the protein was considered the most affirmative region for the ligand binding docking simulation. The docking studies for NVA1309 and mirogabalin were performed without modifying the default parameters. The docking analysis was conducted using Autodock Vina. A grid box size of 70 × 70 × 70 points with spacing of 1.0 Å between the gird points was executed to cover almost the entire favorable protein binding site. The X, Y, Z centers for Cavα2δ-1 were (193.388786, 227.598571, and 205.692500). The docking protocol for rigid and flexible ligand docking comprised 20 independent algorithms. The binding aspect of Cavα2δ-1 residues and their corresponding binding affinity score are regarded as the best molecular interaction. All structure figures were prepared in Discovery Studio

Visualizer and PyMol. The fidelity of the docking model was verified by docking gabapentin and comparing the results to the cryo-EM structure reported in the literature [1].

References

1. Chen, Z., Mondal, A. & Minor, D.L. Structural basis for CaVα2δ:gabapentin binding. Nat Struct Mol Biol 30, 735–739 (2023).

2. BIOVIA Discovery Studio Client v19.1.0.18287. Accelrys Discovery Studio; Accelrys Software Inc.: San Diego, CA, USA, 2019.
